# P-1528. Investigating Patient and Microbiological Factors to Unravel the Path to Septic Shock

**DOI:** 10.1093/ofid/ofaf695.1709

**Published:** 2026-01-11

**Authors:** Christian Hendrix, Andrea M Heredia Castillo, Jennie H Kwon, Andrew Atkinson, Maria Cristina Vazquez Guillamet

**Affiliations:** Washington University, st louis, MO; Washington University School of Medicine in St. Louis, St Louis, Missouri; Northwestern University , Chicago , IL; Washington University School of Medicine, St. Louis, Missouri; Washington University in St. Louis, St.Louis, Missouri

## Abstract

**Background:**

Sepsis is a clinical syndrome with variable progression, where some cases evolve into septic shock. This project aims to identify features -host-related, microbial, or interactions between the two- that drive the progression from sepsis to septic shock.

**Table 1.**
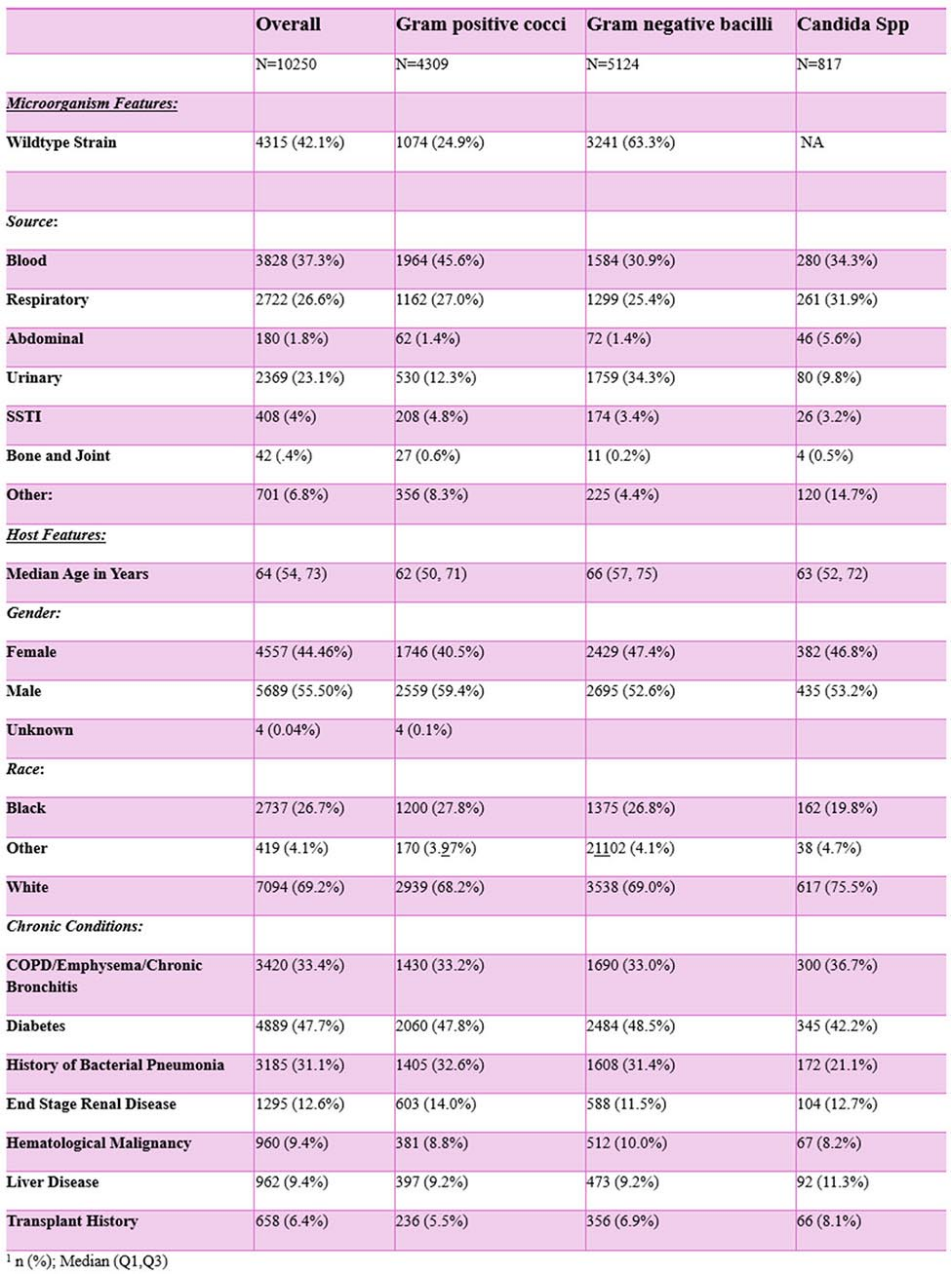
Cohort Description with Host and Microbiological Characteristics Categorized by Microorganism 1 n (%); Median (Q1,Q3). Overall sepsis episodes categorized by microorganism category within individual columns. This table is also organized by microorganism specific factors as well as host factors outlined above within rows.

**Figure 1.**
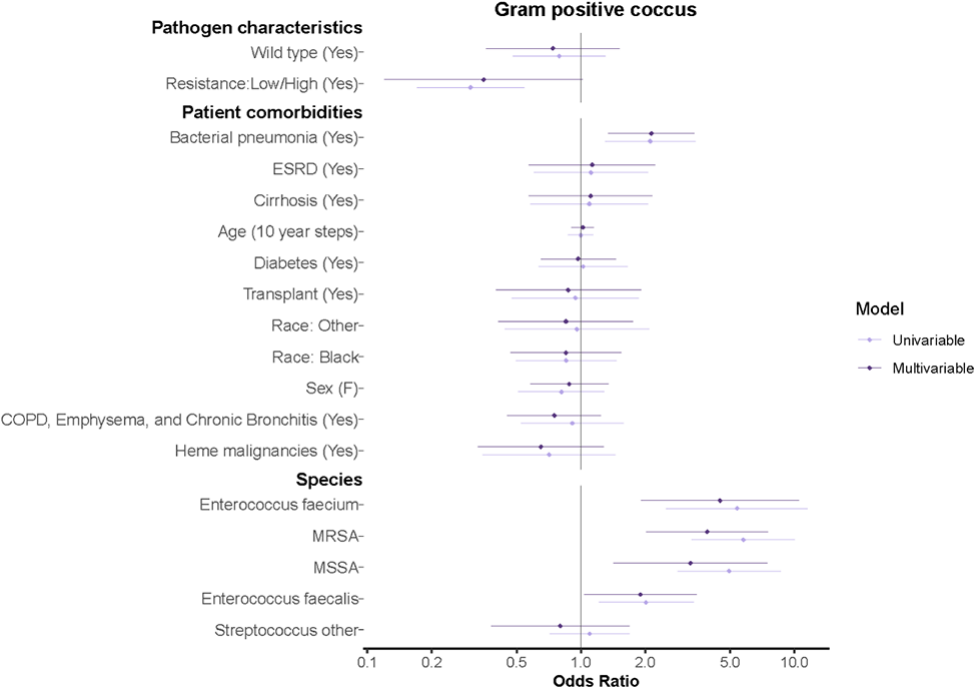
Univariable and Multivariable Generalized Linear Models for Patients with Sepsis Episodes and Gram-Positive Cocci Culture Data Gram-positive cocci (GPC): Enterococcus faecalis, Enterococcus faecium, methicillin-sensitive Staphylococcus aureus, methicillin-resistant Staphylococcus aureus, Streptococcus spp. The outcome of interest in analyzed GPC sepsis episodes was septic shock as a binary variable (0/1). Univariable and multivariable generalized linear models are listed above. The model analyzed pathogen characteristics, patient host comorbidities, and microorganism subtype listed on the y-axis. The model controlled for these factors while using standard errors accounting for multiple measurements per patient host participant. The Odds Ratio is listed on the x-axis.

**Methods:**

This was a retrospective cohort study of patients admitted to Washington University/Barnes-Jewish Hospital from 2016 to 2021. Adult patients with either community- or hospital-onset sepsis as defined by the CDC adult sepsis event criteria and with positive culture were analyzed. Our outcome is the presence of septic shock, defined as ≥12 hours of vasopressor use. Univariable and multivariable generalized linear models were fitted, controlling for patient host factors and microorganism factors, with sandwich-type standard errors calculated to account for potential correlation from multiple measurements per participant. Separate models were fitted for gram-positive cocci (GPC), gram-negative bacilli (GNB), and *Candida spp.*

**Figure 2.**
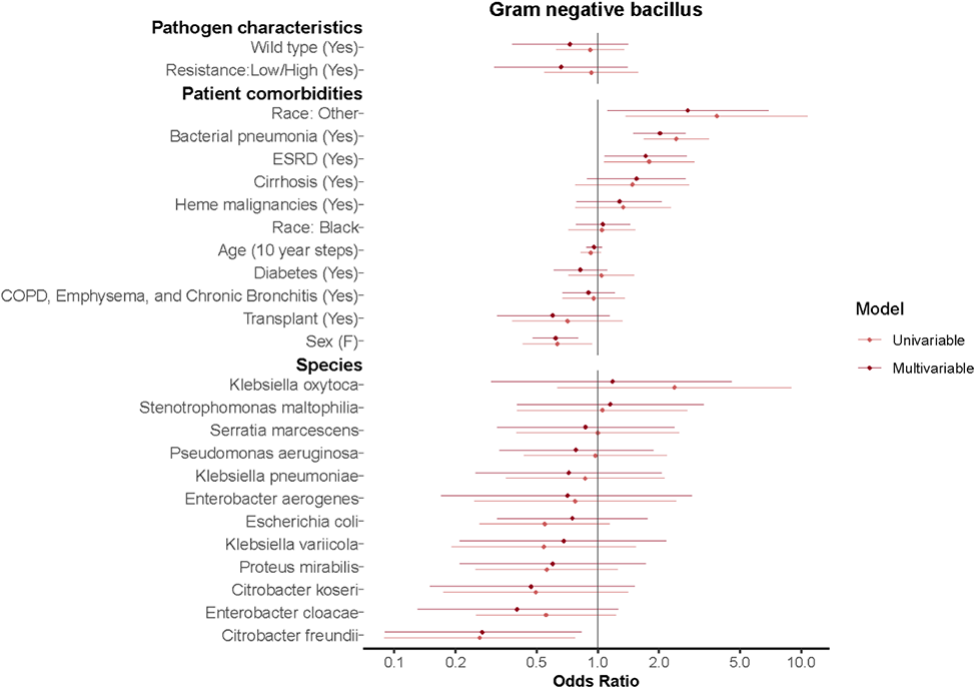
Univariable and Multivariable Generalized Linear Models for Patients with Sepsis Episodes and Gram-Negative Bacilli Culture Data Gram-negative bacillus (GNB) including: Citrobacter freundii, Citrobacter koseri, Enterobacter aerogenes, Enterobacter cloacae, Escherichia coli, Klebsiella oxytoca Klebsiella pneumoniae, Klebsiella variicola, Proteus mirabilis, Pseudomonas aeruginosa, Serratia marcescens, Stenotrophomonas spp. The outcome of interest in analyzed GNB sepsis episodes was septic shock as a binary variable (0/1). Univariable and multivariable generalized linear models are listed above. The model analyzed pathogen characteristics, patient host comorbidities, and microorganism subtype listed on the y-axis. And the model controlled for these factors while using standard errors accounting for multiple measurements per patient host participant. The Odds Ratio is listed on the x-axis.

**Figure 3.**
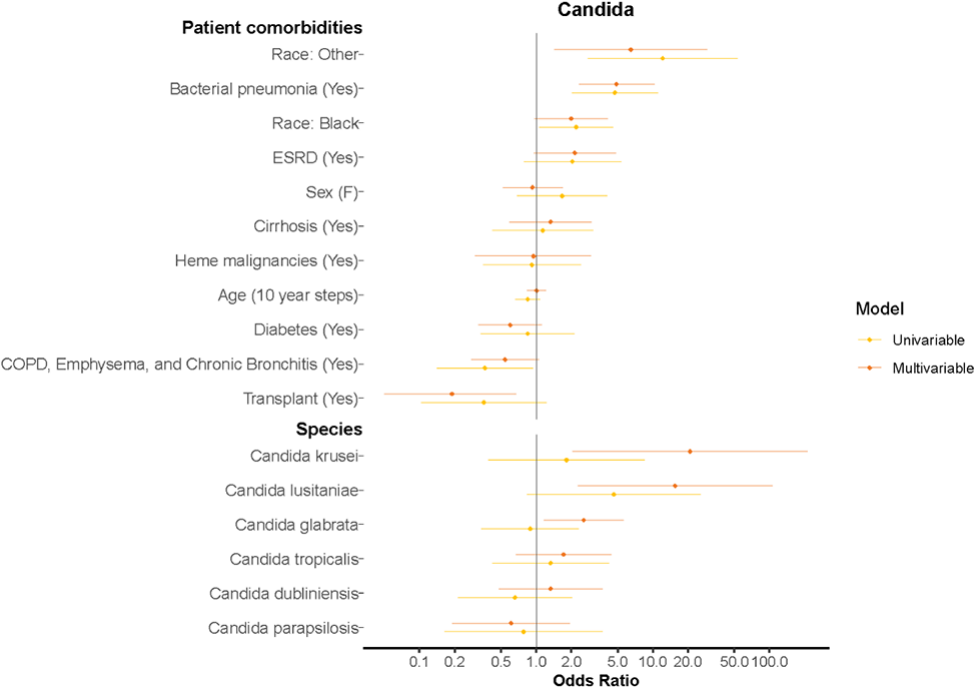
Univariable and Multivariable Generalized Linear Models for Patients with Sepsis Episodes and Candida Spp Culture Data Candida spp including: Candida albicans, Candida dubliniensis, Candida glabrata, Candida krusei, Candida lusitaniae, Candida parapsilosis, Candida tropicalis. The outcome of interest in analyzed Candida spp sepsis episodes was septic shock as a binary variable (0/1). Univariable and multivariable generalized linear models are listed above. The model analyzed pathogen characteristics, patient host comorbidities, and microorganism subtype listed on the y-axis. The model controlled for these factors while using standard errors accounting for multiple measurements per patient host participant. The Odds Ratio is listed on the x-axis.

**Results:**

In total, 38,887 sepsis episodes from 10,250 patients with 16,732 admissions were analyzed. Patient level data by microorganism categories are outlined in Table 1. Median age was 64 years old ([IQR] 54-73). Most positive cultures were blood, followed by respiratory specimens. Of isolated GNB, 63.3% were wildtype. Host features, such as a previous history of pneumonia, contributed to development of septic shock: GPC OR 2.14 (95% CI 1.34-3.40, p< 0.05), GNB OR 2.02 (95% CI 1.50-2.70, p< 0.001), and *Candida spp* OR 4.89 (95% CI 2.32-10.29, p< 0.001). Certain species were more likely to be associated with septic shock, including methicillin-sensitive *S. aureus* OR 3.26 (95% CI 1.42-7.47, p< 0.05), methicillin-resistant *S. aureus* OR 3.90 (95% CI 2.02-7.53, p< 0.001), *E. faecium* OR 4.49 (95% CI 1.91-10.51, p< 0.001), and *C. krusei* OR 20.9 (95% CI 2.05-213.42, p< 0.05). Antimicrobial resistant strains were not associated with septic shock.

**Conclusion:**

This preliminary analysis highlights the importance of previous infections in the progression to septic shock with subsequent infections. It also reveals that antimicrobial resistance was not a marker for severity of presentation in this study.

**Disclosures:**

Maria Cristina Vazquez Guillamet, MD, Bionano: Stocks/Bonds (Public Company)|Charisma: Stocks/Bonds (Public Company)|Ocugen: Stocks/Bonds (Public Company)

